# The effect of geographical variation in income measures on measles-mumps-rubella uptake and coverage in England; a protocol for an ecological study

**DOI:** 10.1371/journal.pone.0280008

**Published:** 2023-06-28

**Authors:** Salma Ghazal, Ireneous N. Soyiri

**Affiliations:** Hull York Medical School, University of Hull, Hull, United Kingdom; University of Rajshahi, BANGLADESH

## Abstract

Measles is a vaccine-preventable disease whose vaccine was introduced in England in 1988, however, Measles outbreaks have still been occurring in the country. Consequently, the World Health Organization (WHO) removed the elimination status of Measles in 2019 from England and the whole United Kingdom. Noticeably, MMR vaccination coverage in England is below the recommended threshold with geographical variations across local authorities (LA). The research into the effect of income disparities on MMR vaccine coverage was insufficiently examined. Therefore, an ecological study will be conducted aiming at determining whether there is a relationship between income deprivation measures and MMR vaccine coverage in upper-tier local authorities in England. This study will be using 2019 publicly available vaccination data for children who were eligible for the MMR vaccine by their second and fifth birthday in 2018/2019. The effect of spatial clustering of income level on vaccination coverage will also be assessed. Vaccination coverage data will be obtained from “Cover of Vaccination Evaluated Rapidly (COVER)”. Income deprivation score, Deprivation gap, and Income Deprivation Affecting Children Index will be obtained from Office for National Statistics and Moran’s Index will be generated using RStudio. Rural/urban LA classification and mothers’ education will be included as possible confounding factors. Additionally, the live births rate per mothers’ age group will be included as a proxy for the mothers’ age variation in different LA. Multiple linear regression will be used after testing the relevant assumptions, using SPSS software. Moran’s I together with income deprivation score will be analysed through regression and mediation analysis. This study will help in determining whether income level is a determinant of MMR vaccination uptake and coverage in LA in England which would help policymakers in designing targeted campaigns, thus preventing measles outbreaks in the future.

## Introduction

The use of Measles, Mumps and Rubella (MMR) trivalent vaccine began in England in 1988 [1]. Two doses of the vaccine are recommended in England, as part of the national immunisation schedule. The first dose is scheduled at the age of 12 months and the second at the age of 3 years and 4 months [2]. However, after more than thirty years of its introduction, measles is not considered something of the past as yet, as several measles outbreaks have been occurring in England during the last decade [3–5]. Consequently, the Measles elimination status was removed by the World Health Organization (WHO) in 2019 after outbreaks of Measles occurred in 2018 [5,6]. In addition to the outbreaks, the overall vaccination coverage of measles in England is below the recommended herd immunity threshold of 95%, with noticeable, significant geographical variations in coverage across different local authorities [7,8].

A local authority in England is a geographical administrative division for local government that have some functions and deliver different services [9,10]. There are two main levels, an upper-tier and a lower tier local authority, where upper-tier local authority has broader responsibilities compared to lower-tier. Each tier is responsible for different functions [9,10]. However, in some areas there is a single tier LA, named unitary authority that would be responsible for all the functions combined. At a lower level than lower-tier LA, there are in some areas, parish, community, or town councils, as well [9,10].

**Fig 1:**
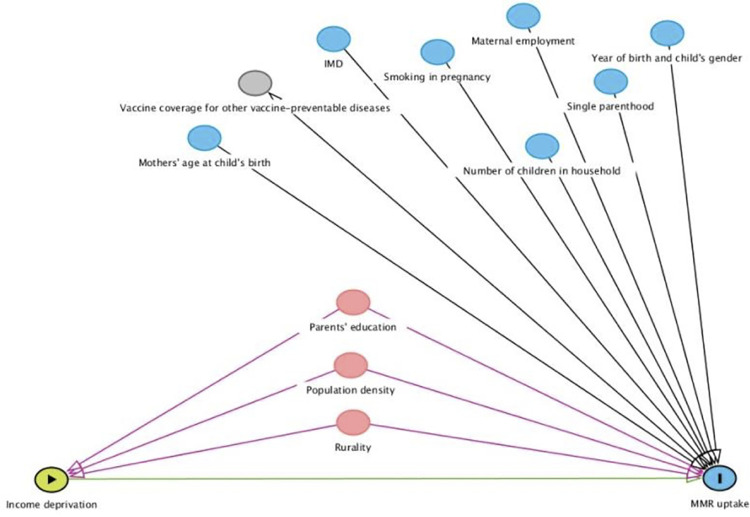
Represents a Directed Acyclic Graph (DAG) showing the factors that were found to be affecting MMR uptake in England (IMD is the Index of Multiple Deprivation). DAG was constructed using Ferguson *et al*, (2020) proposed methods for creating DAGs [19]. The image was generated using DAGitty.net online software [20].

Some studies found a relationship between the decreased uptake of MMR and low household income or unemployment [11–13]. Moreover, mothers’ mental health was also found to be associated with low uptake [14], as well as, mothers’ education level; age when she gave birth to the child; and employment status [15]. Other reported factors that withheld parents from giving their children the vaccine were diverse [16]. Some of them were related to access difficulties faced by some minority groups [17], but other parents had concerns surrounding the safety of the vaccine [15,18]. A Directed Acyclic Graph (DAG) summarising the factors that were found to be associated with MMR uptake is shown in Fig 1 below.

Generally, Social Determinants of Health (SDH) have been found to affect health implicitly [21] and some of them were found to affect vaccination uptake and coverage [11,13,22,23]. However, the association between deprivation and uptake of the MMR vaccine specifically was insufficiently examined in England, and studies on this topic gave inconsistent results [11,15]. Some were conducted on a local level [11] while others were conducted on a national level but used different methodologies and time periods [23]. Therefore, research into the factors that affect the geographical variation of MMR vaccine uptake and coverage in England is needed to be able to tackle this problem by implementing targeted programs.

### Study objectives

The primary objective of the study is to determine whether there is a relationship between income deprivation measures in upper-tier local authorities and MMR vaccine uptake and coverage in England in 2019. The study cohort are children who were eligible for the MMR vaccine by their second and fifth birthday in 2018/2019.

The secondary objective is to determine the effect of spatial clustering of income data on vaccination uptake and coverage.

## Methods

### Study type

Geographical ecological study based on retrospective, publicly available data. The geographical units are upper-tier local authorities in England. The choice of this geographical unit was based on vaccination data which are only available on this geographical level as the smallest unit.

STROBE 2007 (v4) Statement checklist for cross-sectional studies was used for the protocol design and is available in the S2 Table in appendix.

### Timing of final analysis

Outcomes will be analysed collectively, as the data are retrospective and published online prior to the start of the analysis.

### The outcome variables and their calculation

The outcome variables are MMR vaccine uptake by 2 years of age (first dose) and coverage by 5 years of age (second dose) on upper-tier local authority geographical level. Both outcomes are continuous and represented as percentages. The term uptake was used in the protocol to refer to the first dose of MMR vaccine. On the other hand, coverage was used to refer to the percentage of children who received two doses of MMR vaccine, reflecting completeness of the 2 doses. This would help to distinguish between the children who received the first dose only and those who completed the full recommended course and hence determining the possible factors that could have an effect on each of them separately. The whole population vaccination data will be obtained from national statistics which are routinely collected by Public Health England as part of the Cover of Vaccination Evaluated Rapidly (COVER). COVER extracts its data from Child Health Information Systems (CHIS) and from General Practices (GP) systems in a few local authorities [24], which is a good representation of the children population in England.

Vaccine coverage percentages were calculated before the data were publicly published by Public Health England. The calculation was conducted by dividing the number of eligible populations who were immunised in each upper-tier local authority (LA) by the number of eligible populations in that local authority, then multiplied by 100. The eligible population was defined as “the total number of children in the LA responsible population, reaching their nth birthday in the collection year” [24]. The eligible population includes both, children registered with a GP in the local authority and those who are not registered but lived in that LA [24]. The data is available for only 149 upper-tier local authorities, as 3 local authorities’ data were added to other LA [24]. Data were published in September 2019 [7] and covered the period from April 2018 to March 2019 [25].

### The independent variables

Moran’s Index will be included in the analysis as an indicator for spatial clustering of income deprivation in upper-tier LA. The calculation methodology will be based on that adopted by Nyanzu and Rae (2019) using R software [26]. It will involve income deprivation scores in Lower Layer Super Output Areas (LSOA) that constitute each local authority [26]. Moran’s Index equation that will be used for the calculations is shown below [26]:

$$I=\frac{N\Sigma_{i}\Sigma_{j}W_{i,j}(X_{i}-\bar{x})(X_{j}-\bar{x})}{{\left(\Sigma_{i}\Sigma_{j}W_{ij})\Sigma_{i}(X_{j}-\bar{x}\right)}^{2}}$$

Where:

*I* is the Global Moran’s Index;

*N* Is the number of observations.

*X*_*i*_ is the X variable when at area i.

*X*_*j*_ is the X variable when at area j.

$\bar{x}$ is the mean of the X variable.

*Wij* is the spatial weight used to compare area i and area j [26].

Deprivation gap data were published on a lower-tier local authority level, thus their average in each upper-tier local authority will be calculated. These variables were published by the Office for National Statistics and are based on data for the English Indices of Multiple Deprivation, 2019 [27].

The income deprivation score, as well as the Income Deprivation Affecting Children Index (IDACI) 2019 on upper-tier local authority level will be included in the analysis. Data are available for 151 Upper-tier local authorities in England [28]. A higher income deprivation score means that the area is more deprived [28]. The 2019 Deprivation data collected represent the years 2015/2016 and are the latest published data [28].

The percentage of children per ethnic group in each local authority, based on 2011 census will also be included [29]. In addition to the country of birth of individuals who live in families with dependent children [30].

Additionally, the live births rate for mothers’ age group in local authorities will be used as a proxy for the variation in mothers’ age in different local authorities. Data for the year 2016 [31] will be used with the analysis of the uptake outcome variable, and data for the year 2013 [32] will be used for the coverage outcome variable. The percentage of females aged 16–49 years in each category of highest qualification achieved (total 7 categories) in a LA will also be included in the analysis as a measure of variation in education across different local authorities [33]. The total number of females aged 16–49 years who are residents in a local authority, based on the 2011 census will be used as a denominator to calculate the percentage in the corresponding LA [34].

Data are publicly available, free to use, published by the Ministry of Housing, Communities & Local Government and contain public sector information licensed under the Open Government Licence v3.0 [35].

#### Covariates and confounding factors

To adjust for possible confounding factors, the following variable will be added to the multiple linear regression analysis. This is rural/urban classifications of upper-tier local authorities [36]. This variable will be recoded using the dummy method to allow it to be included in the multiple linear regression. This was previously considered as a confounding factor in previous studies and was found to have an effect on MMR vaccine uptake [23].

### Study population and data collection

The study population is children living in England who were eligible for the MMR vaccine by their second and fifth birthday in 2018/2019 and who were registered with a GP in an upper-tier local authority or those who lived in the corresponding upper-tier local authority if they were not registered with a GP [25]. Data will be collated from datasets published by the aforementioned sources and linked by upper-tier local authority’s codes and names in a new Excel spreadsheet.

## Data analysis

### Assumptions’ testing

Our initial choice of modelling the outcome variable will be to use a Linear Regression, hence we will test for the key assumptions of simple linear regression. The residuals of each outcome variable will be tested for normality visually by assessing a Q-Q plot and statistically by the Shapiro-Wilk test [37] at a significance level of 0.05, where the null hypothesis would be that the sample distribution is normal. If the result is significant, then the distribution will be regarded as non-normal.

The homoscedasticity assumption will also be tested before conducting the linear regression by plotting the standardised residuals against the standardised predicted values on a scatterplot. Moreover, the independent errors’ assumption will also be tested by plotting the standardized residuals against standardized predicted values. Randomly dispersed data with a rectangle shape and values lying between -3 and 3 on both the y and x-axes will be regarded as independent. Linearity will also be assessed for each independent variable versus each outcome by plotting them on a scatterplot.

We will consider other statistical approaches to transforming the outcome variable if we determine that it is not normally distributed for example by log-transformation. Alternatively, we would also consider adopting a count model, such as Poisson or Negative binomial regressions.

Finally, for multiple linear regression, Cook’s distance will be used to determine whether there are outliers that can influence the model and introduce bias, as well as the multicollinearity of independent variables through the Variance Inflation Factor (VIF) method and correlation coefficients.

### Summary statistics

If the data are normally distributed, description of the location and dispersion of outcome variables will be described by mean, and Standard Deviation (SD). Otherwise, the median, and interquartile range will be described. Data will be represented using a box-and-whisker plot.

Percentages will be used for categorical data as a summary statistic and the number of final local authorities to be included will also be described.

### Statistical tests

A two-sided significance level will be set at 0.05 for all relevant statistical tests.

Simple Linear Regression will be conducted after testing for its assumptions as previously mentioned.

A regression coefficient will be determined, as well as R^2^ and adjusted P-value. A R^2^ > 70% will be considered to have a strong effect size, whereas, an R^2^ between 50% and 70% will be considered to have a moderate effect size, as adopted by Brennan, Moore and Millar (2022) [38].

Afterwards, multiple linear regression for the independent variables that were found to be associated with the outcome will be conducted after testing the relevant additional assumptions mentioned earlier. The standardized coefficients will be compared, in addition to the adjusted R^2^ and the P-value. Cook’s distance values greater than 1 will also be reported. Both statistical analyses will be conducted for both outcome variables.

Mallows Cp will be used to evaluate regression models. A value less than or equal to the number of parameters in any suggested model will be regarded as an unbiased model except for the full parameters model which will not be evaluated based on Mallow’s Cp. Bayesian Information Criterion (BIC) and Akaike Information Criterion (AIC) will also be used as complementary methods to Mallow’s Cp for the evaluation of regression models. The models will be compared and the ones with the lowest values for BIC and AIC will be shortlisted.

To analyse the secondary outcome, if the income deprivation score and Moran’s index are found to be associated with the outcomes, then mediation analysis will be conducted. Bootstrapping will be conducted afterwards to test for mediation effect significance.

An alternative method if the outcome variables’ distribution is non-normal is Generalized Linear Model (GLM). The independence of outcome variables’ observations will be checked before performing it, as well as determining the type of distribution and link function of the data.

Finally, sensitivity analysis will be conducted to assess the sensitivity of outcome variables to confounding factor and outliers.

### Handling missing data

Six Local authorities will be excluded from the analysis. These are the City of London, Hackney, Rutland, Leicestershire, Cornwall, and the Isles of Scilly. This is because MMR coverage data for the City of London was reported under Hackney. Also, data for Rutland were reported under Leicestershire, in addition to Cornwall which contains data for the Isles of Scilly. The merge of two LA vaccination coverage data into one would affect the results of the analysis, taking into consideration the significant differences in income deprivation scores between them. For instance, the income deprivation rank of average score in the City of London is 144 and that of Hackney is 17, where 1 is the most deprived and 151 is the least deprived.

### Statistical packages

The data will be analysed using SPSS and Stata software, as well as RStudio

2022.07.1+ 554.

### Ethics

Ethical approval will not be required for this research because it will use publicly available data and does not seek to work with or identify personal identifiers.

### Strengths and limitations

To begin with, this study’s potential strengths, lie in its focus on tackling the re-emergence of MMR and trying to determine the factors that could have an impact on MMR uptake and coverage in England which in turn could have resulted in the removal of the elimination status of measles and rubella by the World Health Organization in 2019. It also aims to determine whether spatial clustering of income level influences the uptake and coverage of MMR vaccine in England.

On the other hand, a limitation of this work would be the use of aggregated data on upper-tier local authority level, meaning that further individual-level studies /research is required on this topic before being able to generalize the results.

### Dissemination

The results of the study would be discussed with key stakeholders through Personal and Public Involvement (PPI). This would be facilitated by the Hull York Medical School’s PPI coordinator. It is also intended to be disseminated through peer-reviewed journals and other media coverage.

## Supporting information

S1 Table Data dictionary. (DOCX)

S2 Table STROBE checklist. (DOCX)
