# Supplementary material for: The effect of geographical variation in income measures on measles-mumps-rubella uptake and coverage in England; a protocol for an ecological study
Source: PLoS One. 2023 Jun 28;18(6):e0280008. doi: 10.1371/journal.pone.0280008 (PMC10306216; doi:10.1371/journal.pone.0280008)
Supplement: S1 Table — (DOCX) [file pone.0280008.s001.docx]

**Supporting information:**

## **Appendix**

## **S1 Table:** Data dictionary

| **Variable** | **Variable name** | **Measurement unit** | **Allowed values** | **Variable definition** | **Description** | **References** |
| --- | --- | --- | --- | --- | --- | --- |
| LA_code | Local authority code | ID  String | E06000001 - E10000034 | Upper-tier local authority code. | A unique code assigned to each Upper-tier LA. | [28] |
| LA_name | Local authority name | ID  String | text | Upper-tier local authority name. | A unique name given to each Upper-tier LA. | [28] |
| MMR_uptake | MMR vaccine uptake | Numeric  Percentage | 74.3 to 97.1% | The percentage of MMR vaccine uptake by 2 years of age (first dose) in upper-tier local authorities. | The number of eligible populations who were immunised by their second birthday in each upper-tier local authority (LA) divided by the number of eligible populations in that local authority x 100. | [7] |
| MMR_coverage | MMR vaccine coverage | Numeric  Percentage | 64.1 to 96.4% | The percentage of MMR vaccine coverage by 5 years of age (second dose) in upper-tier local authorities. | The number of eligible populations who were immunised with 2 doses of the MMR vaccine by their fifth birthday in each upper-tier local authority (LA) divided by the number of eligible populations in that local authority x 100. | [7] |
| MoransI | Moran’s Index | Numeric  Score | -1 to 1 | It is a measure of spatial autocorrelation, measuring the clustering of income deprivation. | 1 Perfect clustering  -1 Perfect dispersion  0 Randomness | [26] |
| Deprivation_gap | Deprivation gap | Numeric  Percentage | 8.4 - 56.7% | It is the difference between the maximum 'Income Score’ and the minimum 'Income Score' in each lower-tier local authority. | An average deprivation gap in upper-tier LA is calculated for this variable. | [27] |
| Income_deprivation_score | Income deprivation average score | Numeric  Average score | 0.029 to 0.251 | A score based on combining the values returned from indicators related to income. | Income average score per upper-tier local authority. It is calculated by compiling data from LSOAs (population-weighted averages). Areas with the highest scores are the most deprived. | [28] |
| IDACI | Income Deprivation Affecting Children Index (IDACI) | Numeric  Proportion | 0.032 to 0.327 | The proportion of children aged 0-15 years, who live in income-deprived families. | A proportion. | [28] |
| Rural_urban | Rural/urban classification of upper-tier local authorities | Categorical data  (3 categories) | \| Predominantly Urban \| \| --- \| \| Urban with Significant Rural \| \| Predominantly Rural \| | The rural/ urban classification of upper-tier local authorities is based on the percentage of the population living in rural areas. | The classification is based solely on the percentage of the population residing in rural areas in the specified geographical unit. Predominantly rural is where ≥ 50% of the population lives in rural areas. Predominantly urban is where ≥ 74% of the population reside in urban areas, whereas, urban with significantly rural is where 26 to 49% of the population live in rural areas. | [39] |
| Births_rate2016 | Live births rate per mothers’ age group in 2016 | Numeric  Rate | 0.3-171.3 | Live births rate per 1000 women per age group in every LA, in 2016. | Rate per 1000 women.  The age groups of mothers at birth are under 20, 20-24, 25-29, 30-34, 35-39, 40-44, 45 and over. | [31] |
| Births_rate2013 | Live births rate per mothers’ age group in 2013 | Numeric  Rate | 0.2-169.9 | Live births rate per 1000 women per age group in every LA, in 2013. | Rate per 1000 women.  The age groups of mothers at birth are under 20, 20-24, 25-29, 30-34, 35-39, 40-44, 45 and over. | [32] |
| Education | The highest level of qualification achieved. | Numeric  Percentage | 0.216 - 71.482% | The percentage of females aged 16 to 49 years per each (highest) level of qualification achieved in a local authority per total female population aged 16-49 years in that LA. | The levels of qualifications are:   - No qualifications; - Level 1: 1-4 O Levels/CSE/GCSEs (any grades), Entry Level, Foundation Diploma, NVQ Level 1, Foundation GNVQ, Basic/Essential Skills; - Level 2: 5+ O Level (Passes)/CSEs (Grade 1)/GCSEs (Grades A*-C), School Certificate, 1 A Level/ 2-3 AS Levels/VCEs, Intermediate/Higher Diploma, Welsh Baccalaureate Intermediate Diploma, NVQ level 2, Intermediate GNVQ, City and Guilds Craft, BTEC First/General Diploma, RSA Diploma; - Apprenticeship; - Level 3: 2+ A Levels/VCEs, 4+ AS Levels, Higher School Certificate, Progression/Advanced Diploma, Welsh Baccalaureate Advanced Diploma, NVQ Level 3; Advanced GNVQ, City and Guilds Advanced Craft, ONC, OND, BTEC National, RSA Advanced Diploma; - Level 4 and above: Degree (for example BA, BSc), Higher Degree (for example MA, Ph.D., PGCE), NVQ Level 4-5, HNC, HND, RSA Higher Diploma, BTEC Higher level, Foundation degree (NI), Professional qualifications (for example teaching, nursing, accountancy); - Other qualifications: Vocational/Work-related Qualifications, Foreign Qualifications (not stated/level unknown). | [33] |
| Ethnicity | Ethnicity | Numeric  Percentage | 0 - 97.152% | Percentage of dependent children in each ethnic group in each local authority by ethnic group of Household Reference Person. | The ethnic groups are:   - White_English/Welsh/Scottish/Northern Irish/British - White_Irish - White_Other White (also includes Gypsy or Irish Traveller) - Mixed/multiple_ethnic_group (includes White and Black Caribbean, White and Black African, White and Asian, Other Mixed) - Asian/Asian_British - Black/African/Caribbean/Black_British - Other_ethnic_group (includes Arab and Any other ethnic group) | [29] |
| Country_of_Birth | Country of Birth of individuals in families with dependent children. | Numeric  Percentage | 0.03 – 97.73% | Number of individuals who live in families with dependent children per category of country of birth. | The countries of birth were grouped into the following 6 categories:   - United Kingdom. - Rest of Europe. - Africa. - Middle East and Asia. - The Americas and the Carribbean. - Antarctica, Oceania (including Australasia) and other. | [30] |
